# Minimally invasive treatment of benign oral vascular lesions: A retrospective study

**DOI:** 10.4317/medoral.26809

**Published:** 2025-02-15

**Authors:** Herberth Campos Silva, Ana Cláudia Oliveira Teles, Gabriela Fonseca Rocha, Esmeralda Maria da Silveira, Dhelfeson Willya Douglas-de-Oliveira, Ana Terezinha Marques Mesquita

**Affiliations:** 1 Department of Dentistry, Faculty of Biological and Health Sciences, Federal University of the Jequitinhonha and Mucuri Valleys, Diamantina, Minas Gerais, Brazil

## Abstract

**Background:**

The ethanolamine oleate (EO) in different concentrations has been used in sclerotherapy of oral benign vascular lesions (OBVLs). The aim of the present study was to define demographic and clinical characteristics of patients with OBVLs treated with 5% EO.

**Material and Methods:**

A retrospective study was conducted of cases treated by sclerotherapy from 1992 to 2022, and medical records of 52 patients with OBVLs were analysed. Thus, 44 cases with complete data were selected and described. Categorical data were analyzed using the chi-square test, with the significance level set at 5% (*p* < 0.05).

**Results:**

Mean age was 52.89 years (range seven to 82 years). The female sex was predominant (77%) and brown was the most common skin color (54%). Most lesions occurred on the lower lip (65.9%), tongue (11.4%), buccal mucosa (9.1%), upper lip (6.8%) and palate (25%.3%). Approximately 73% of the lesions were ≤ 1 cm, > 1 ≤ 2 cm (18.2%,) and > 2 cm (9.1%). Complete regression of the lesions occurred in 97.7% of the cases, with no case of recurrence and no need for complementary surgery. All patients were satisfied with the treatment.

**Conclusions:**

Sclerotherapy with 5% EO is a safe and effective method for the treatment of OBVLs.

** Key words:**Hemangioma, vascular malformations, sclerotherapy, ethanolamine oleate.

## Introduction

Hemangioma and vascular malformations are benign vascular lesions that are common in the head and neck region. The International Society for the Study of Vascular Anomalies classifies vascular anomalies as proliferative vascular lesions and non-proliferative vascular malformations (1). This lesions appears as a red, purplish-red or purplish spot, nodule or papule, depending on the degree of congestion and depth of the lesion in the tissue, with higher incidence in the female sex, individuals with white skin color and premature newborns (2). In most cases, these lesions are not present at birth, develop in three phases (proliferation, involution and involuted) and tend to have a firm, rubbery consistency and well-defined limits (3).

Vascular malformations (VMs) can occur in arteries, veins and/or capillaries, are considered structural anomalies of embryonic development, affect both females and males, and the incidence in the head and neck region ranges from 14% to 65% (4-6). VMs are characterized as a defect in the maturation of the vessels and vascular morphogenesis caused mainly by the dysregulation of the pathways of embryogenesis and vasculogenesis (7), and are divided into two groups: low and high flow. Low flow VMs consist of a venous, capillary or lymphatic component, whereas high-flow VMs consist of an arterial or arteriovenous component (8,9). Clinically, VMs are similar to hemangioma, but are always present at birth growing with the physical development of the individual, remaining sTable after the growth phase, and do not regress spontaneously (2,3).

Age is a predisposing factor in oral varicosities (VOs), which are often found in individuals older than 60 years of age. VOs are acquired variations of normality characterized by a long, abnormal vein with loosening of the tissue, and increase in venous pressure. Oral varicosities are the most common type and appear as single or multiple bluish-purple papules or nodules on the ventrolateral border of the tongue. VOs can also occur at lower frequencies on the lips and other regions of the oral mucosa, and may require interventions in some cases (10).

The diagnosis of oral benign vascular lesion (OBVL) is based on the clinical history, physical examination and imaging methods when necessary (9). Diascopy is an important auxiliary procedure in the establishment of the differential diagnosis (11). The compression of the glass slide momentarily blanches the lesion and diminishes the size due to vascular emptying, with the subsequent return to it original volume after the removal of the slide, thus excluding the hypotheses of pigmented lesions (3). Aspiration of the lesion contributes to the diagnosis and determination of the hemodynamics (high-flow or low-flow lesion), which is an important factor in the choice of the ideal treatment (12). Indications for interventions in cases of OBVL include esthetic problems, pain, ulcerations, tissue deformation, difficulties with regards to psychosocial relationships and the compression of vital organs with the impairment of function (13).

The most widely used treatments for OBVLs are sclerotherapy, isolated surgery or surgery combined with sclerotherapy, systemic corticosteroids, interferon-α, cryotherapy, radiotherapy, embolization and laser therapy. Sclerotherapy stands out among the different therapeutic options (2), and consists of the intralesional injection of sclerosing agents, causing inflammation in the vessels, followed by vascular occlusion and sclerosis resulting in the regression of the lesion (14). This conservative, noninvasive, low-cost method is effective and does not pose a risk of hemorrhage (15). The different sclerosing agents described in the literature include ethanolamine oleate (EO), absolute ethanol, sodium tetradecyl sulfate and polidocanol (3).

When choosing a sclerosing agent, one must be attentive to its effectiveness, local toxicity, ease of administration and satisfactory results. EO meets these criteria for the treatment of OBVLs (16,17), and is one of the safest and most readily available sclerosing agents. EO is considered effective for the treatment of localized vascular lesions in different regions of the body, including the oral cavity (17). EO is a salt of an unsaturated fatty acid that serves as a sclerosing agent, causing irritation followed by an inflammatory response and fibrosis in the endothelium. It also crosses the vascular wall, causes an extravascular inflammatory response and results in venous sclerosis (18). The oleic acid portion produces inflammation and can activate clotting through the activity of factor XII, which is also known as Hageman factor (2). The ethanolamine portion inhibits the formation of the fibrin clot by the chelation of calcium (18). So, through this mechanism the EO promotes the regression of the lesion replacing the vascular tissue with fibrosis (6,16).

Sclerotherapy with 5% EO is a minimally invasive treatment compared to other methods, which are much more invasive and have both greater risks and higher costs. The lower number of appointments, predicTable results and the possibility of performing the procedure in an outpatient clinic makes sclerotherapy with 5% EO an excellent treatment option for OBVLs (2,17).

The aims of this study were to perform a retrospective analysis of OBVLs treated with sclerotherapy using the intralesional injection of 5% EO, define the demographic characteristics of the patients, identify clinical aspects of OBVLs, side effects and the need or non-need for complementary surgery. In addition, assess the effectiveness of the method and investigate the degree of patient satisfaction with treatment contributing to the establishment of a well-defined, predicTable treatment protocol for OBVLs.

## Material and Methods

The present study was conducted in accordance with the ethical precepts stipulated in the Declaration of Helsinki, and received approval from the Human Research Ethics Committee of Federal University of the Jequitinhonha and Mucuri Valleys (UFVJM) (approval certificate number: 5.960.085). A retrospective study was conducted using data from the records of patients treated at the UFVJM Stomatology Clinic with the diagnosis of OBVLs treated with sclerotherapy in the period from 1992 to 2022. All records selected had a clinical diagnosis of hemangioma, oral vascular malformations or oral varicosities. Records with incomplete data and cases with other types of treatment were excluded. The demographic variables of interest were age, sex and skin color. Data were also collected on the lesion site, size of the lesion, quantity of sclerosing agent used throughout treatment, number of applications, interval of weeks between applications, classification of pain, side effects and adverse effects. Other data of interest were the complete or non-complete regression of the lesion, the need or non-need for complementary surgical treatment, the recurrence or non-recurrence of the lesion, and patient satisfaction with treatment.

The protocol for the treatment of OBVLs was sclerotherapy with an intralesional injection of 5% EO (Ethamolin®, Zest Pharma Ltda., Rio de Janeiro, RJ). The patients were previously informed about the treatment as well as possible discomfort and side effects during and after the administration of the medication. Patients who agreed to the procedures signed a statement of informed consent. Lesion size was measured prior to the administration of the medication. The dose of the sclerosing agent was calculated based on the size of the lesion. For lesions less than 1 cm, up to 0.3 mL of 5% EO was applied at a single injection point. For larger lesions, up to 0.3 mL was applied at three injection points for better distribution of the medication. One mL of 5% EO was slowly applied to the periphery of the lesion with the aid of an insulin syringe. An analgesic was prescribed for two days in case of pain. The return appointment was scheduled for 14 days after the procedure for the assessment of the regression of the lesion (size was measured before, during and after treatment) and the assessment of the need or non-need for further applications of 5% EO. Data on sclerotherapy were recorded on a specific form.

Lesion size was the dependent variable, for which three categories were considered: ≤ 1 cm; > 1 and ≤ 2 cm; and > 2 cm. The independent variables were the final volume of sclerosing agent, number of applications, interval between applications, side effects, regression of the lesion, need for complementary surgery, comorbidities, recurrence of the lesion and patient satisfaction with treatment.

For statistical analysis, the results were organized in a database, with the coding, entering and editing of the data. Data were analyzed with the aid of the Statistical Package for the Social Sciences (SPSS®, version 25). Descriptive statistics was performed for the calculation of absolute and relative frequencies. Categorical data were analyzed using the chi-square test, with the significance level set at 5% (*p* < 0.05).

## Results

A total of 6,668 patient records from the study period (1992 to 2022) were analyzed. Among these, 52 cases of OBVLs were selected and sclerotherapy was the treatment of choice in 44 patients. Average age was 52.89 years (range: seven to 82 years) and women predominated (n = 34; 77.3%) over men (n = 10; 22.3%). In terms of skin color, the majority of the patients were brown (n = 24; 54.5%), followed by white (n = 14; 31.8%) and black (n = 6; 13.6%). Most lesions were on the lower lip (n = 29; 65.9%), followed by the tongue (n = 5; 11.4%), buccal mucosa (n = 4, 9.1%) and upper lip (n = 3; 6.8). Lesion sizes were ≤ 1 cm (n = 32; 72.7%), > 1 cm and ≤ 2 cm (n = 8; 18.2%) and > 2 cm (n = 4; 9.1%) (Table 1).

The analysis of the amount of sclerosing agent used throughout treatment for lesion regression revealed that 0.3 mL was satisfactory in 23 cases (52.3%), amounts from > 0.5 mL to ≤ 1 mL were used in 11 cases (25.0%), amounts from > 0.3 mL to ≤ 0.5 mL were used in seven cases (15.9%) and amounts greater than 1.0 mL were required in three cases (6.8%). A positive correlation was found between lesion size and the amount of sclerosing agent used in the treatments (*p* = 0.030). Two applications of EO were sufficient in most cases (n = 16; 36.5%), one and three applications were sufficient in 11 cases each (50.0%) and four, five or six applications were necessary in two cases each (13.5%). The time between applications of the sclerosing agent was two weeks in 27 cases (61.4%), four or more weeks in 13 cases (29.5%) and three weeks in four cases (9.1%). A positive correlation was found between the number of applications and lesion size (*p* = 0.022; Table 2).

Side effects were reported in 37 cases (84.1%), the most common of which was pain (n = 34; 77.3%), followed by a burning sensation (n = 20; 45.5%), bleeding (n = 13; 29.5%) and ulceration (n = 3; 6.8%). Some patients reported more than one side effect. Mild pain was the most common (n = 14; 31.8%), followed by severe pain (n = 11; 25.0%) and no pain (n = 8; 18.2%). No patient reported unbearable pain. No statistically significant correlation was found between pain and lesion size (*p* = 0.270; Table 2).

**Figure 1:**
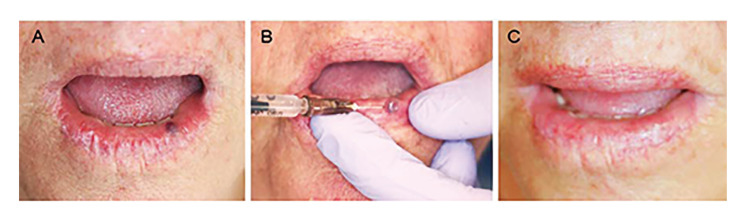
Female patient, 62 years old, with a vascular lesion on the lower lip present for 2 years. A: Initial clinical appearance of the lesion. B: Intralesional application of EO. C: Final clinical appearance showing complete regression of the lesion after a single application of 5% EO.

**Figure 2:**
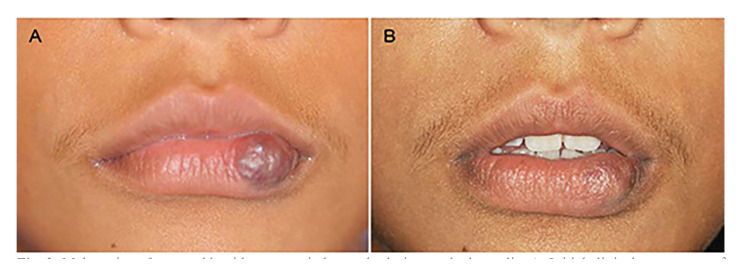
Male patient, 8 years old, with a congenital vascular lesion on the lower lip. A: Initial clinical appearance of the lesion. B: Final clinical appearance showing complete regression of the lesion after six applications of 5% EO.

**Figure 3:**
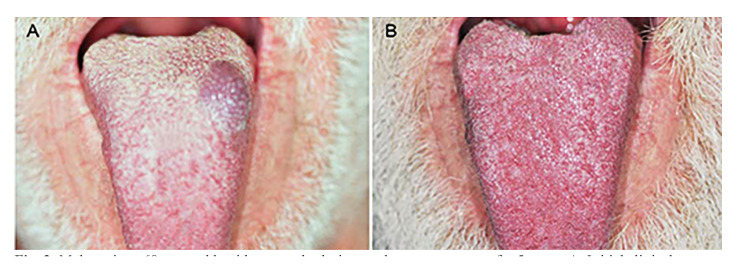
Male patient, 68 years old, with a vascular lesion on the tongue present for 5 years. A: Initial clinical appearance of the lesion. B: Final clinical appearance showing complete regression of the lesion after three applications of 5% EO.

Complete regression of the lesions occurred in 43 cases (97.7%), as illustrated in the Fig. 1, Fig. 2 and Fig. 3; while partial regression occurred in only one case (2.3%). No statistically significant correlation was found between regression (complete or partial) and lesion size (*p* = 0.825). No case of recurrence was found, and there was no need for complementary surgery (Table 2).

## Discussion

Most studies on OBVLs do not clinically differentiate hemangioma and oral vascular malformations or only classify oral vascular malformations as a histological type of hemangioma denominated arteriovenous hemangioma (8,19). In the present study, 95% of cases were clinically diagnosed as hemangiomas or vascular malformations, similar to results reported by Fernandes *et al*. (15), and differs from reports in other studies that describe a high percentage of lesions classified as oral varicosities (17,19). This divergence in epidemiological data remains to this day, even with the classification proposed by Mulliken and Glowack (8), and its acceptance by the International Society for the Study of Vascular Anomalies since 1996. The similarity in the clinical characteristics of hemangiomas and vascular malformations makes the diagnosis difficult. Despite this epidemiological divergence in the literature, the classification of these lesions does not affect the planning and proper treatment of the patients (20,21).

In the present study, OBVLs were identified in seven-year-old children up to 82-year-old individuals and the mean age was 52.89 years. Similar findings have been described in previous studies (1,15). The predominance of the female sex (77.3% of cases) is in agreement with data described in other studies (15,18). In terms of skin color, most patients were brown or white (54% and 31%, respectivelly). In contrast, other studies found a predominance of Caucasians (15) or individuals of African descent (22).

The location of the lesion is a relevant characteristic when defining ideal treatment, as the esthetic factor is a common complaint among patients due to psychosocial discomfort and difficulties in interpersonal relationships (23). In the present study, the most common site of the lesions was the lips, with 29 cases on the lower lip and three cases on the upper lip, which together accounted for 73% of the cases of OBVLs. These results are similar to findings in previous studies (2,19,22). However, other study reported that the tongue was the most affected site (21).

Most of the lesions were ≤ 1 cm, similar to other studies (2,22). When treated with 5% EO, satisfactory results were achieved with a small dose of the medication in cases of smaller lesions (17,24). In the present study, the average number of applications per patient was 2.41, which is lower than the 3.07 applications per treatment with concentrations of 1.25% and 2.5% described in a previous study (2). Kato *et al* . (17) demonstrated that a concentration of 5% EO requires a smaller volume and lower number of applications compared to concentrations of 1.25% and 2.5%. The reduction in the number of applications favors a shorter treatment time and consequently lowers the possibility of patients withdraw from treatment (1,2). The present results in terms of the volume and number of applications of 5% EO were similar to those described in previous studies that also used this same concentration (15,22,24).

Although sclerotherapy is one of the most advantageous treatments, the concentration of the sclerosing agent, dose and administration mode are not standardized in the literature (2,21). Although EO is widely used for the treatment of vascular lesions in other parts of the body, further studies on the treatment of such lesions in the oral cavity are needed. Moreover, most studies published consist of case reports involving different sclerosing agents with different concentrations (17,25).

The 5% EO can cause systemic side effects, such as an anaphylactic reaction and renal toxicity associated with hemolysis and intravascular hemoglobinuria (26). Local side effects include ulcers and necrosis (1,2). These effects occur when the administration of 5% EO is performed directly into the surrounding connective tissue or when vascular leakage occurs due to excessive injection pressure (6). Therefore, slow injection into the lesion is essential to successful treatment with 5% EO (1,15). The margin of safety and reduction of risk is ensured with 1 mL of 5% EO (27). In the present study, we used a dose greater than 1.0 mL on larger lesions in only three cases (6.8%) and no systemic side effects or necrosis were observed.

The option for intervention or follow-up depends on the patient’s age, lesion size and site (3,6) and more than one technique may be necessary for the treatment of vascular lesions (11,15). Laser therapy is indicated for smaller lesions and may cause atrophy in the skin, temporary or permanent hyperpigmentation, mild skin depression or scars (16). Embolization requires sophisticated methods, and is not possible in some cases (3). Surgery can eliminate the lesion, but cannot be performed on all vascular lesions, such as broad diffuse hemangiomas, or in locations of difficult access (2,15). Besides being expensive, surgery poses risks, takes more time and may even require admission to hospital (28). Sclerotherapy is simple, fast and easy to execute, has good tolerability and low cost and can be performed in a dental office (24). In the present study, complete regression of the lesion was achieved in 97.7% (n = 43) of cases, which is compatible with findings described in previous studies (1,24), making surgery, when necessary, only for the removal of the residual fibrotic tissue and phleboliths (3,16). In the present study, complementary surgery was not required in any of the cases. The literature reports that the possibility of complications and side effects cannot be discarded or overlooked (29). Such complications depend on the quality and quantity of the sclerosing agent, lesion size and experience of the clinician (24,30).

There is no consensus in the literature on the EO administration procedure performed with or without anesthesia (22). Most patients in the present study reported some degree of pain (77.3%) during the procedure without anesthesia irrespective of lesion size (Table 1). Other reported side effects were a burning sensation, bleeding and ulceration in 54% (n = 24), 29% (n = 13) and 7% (n = 3) of cases, respectively. The same side effects have been reported in previous studies that performed sclerotherapy with anesthesia (15,24). These data underscore the importance of care, mastery over the administration technique and professional experience in the treatment of OBVLs.

In the present study, OBVLs were more common on the lips, in women and in individuals with brown skin; mean age of the patients was approximately 53 years and most lesions were ≤ 1 cm in diameter. In conclusion, sclerotherapy with 5% EO is a minimally invasive, highly efficient treatment method that achieves the complete regression of lesions in up to 98% of cases of OBVLs. In addition, requires a smaller number of applications, has few side effects and is related to a high degree of patient satisfaction. Thus, the protocol used at our institution for more than 30 years for the treatment of OBVLs with 5% EO is safe and effective and favors a shorter treatment time.

## Figures and Tables

**Table 1:** Demographic characteristics, pain intensity and regression in the cases studied of OBVLs.

Variable		Size						*p-value*
		≤ 1 cm		>1 and ≤ 2 cm		> 2 cm		
		n	%	n	%	n	%	
Sex	Female	26	81.3	6	75.0	2	50.0	0.367
	Male	6	18.7	2	25.0	2	50.0	
Skin color	Brown	19	59.4	3	37.5	2	50.0	0.732
	White	9	28.1	4	50.0	1	25.0	
	Black	4	12.5	1	12.5	1	25.0	
Vascular lesions	Hemangioma/VM	31	96.9	7	87.5	4	100.0	0.471
	Oral varicosities	1	3.1	1	12.5	0	0.0	
Site	Lower lip	23	71.9	5	62.5	1	25.0	0.034
	Upper lip	3	9.4	0	0.0	0	0.0	
	Tongue	2	6.3	1	12.5	2	50.0	
	Palate	1	3.1	0	0.0	0	0.0	
	Buccal mucosa	3	9.4	0	0.0	1	25.0	
	Other	0	0.0	2	25.0	0	0.0	

VM: Vascular malformations.

**Table 2:** Data related to oral benign vascular lesions. Legends.

Variable		Size						*et al.*
		≤ 1 cm		>1 and ≤ 2 cm		> 2 cm		
		n	%	n	%	n	%	
Quantity of sclerosing agent	≤ 0.30 ml	21	65.6	0	0.0	2	50.0	0.030
	> 0.30 ml and ≤ 0.50 ml	4	12.5	3	37.5	0	0.0	
	> 0.50 ml and ≤1.00 ml	6	18.8	4	50.0	1	25.0	
	> 1.00 ml	1	3.1	1	12.5	1	25.0	
Number of applications	1	10	31.3	0	0.0	1	25.0	0.022
	2	12	37.5	2	25.0	2	50.0	
	3	9	28.1	2	25.0	0	0.0	
	4	0	0.0	2	25.0	0	0.0	
	5	0	0.0	1	12.5	1	25.0	
	6 or more	0	0.0	1	12.5	1	25.0	
Interval between applications	2 weeks	22	68.8	2	25.0	3	75.0	0.033
	3 weeks	4	12.5	0	0.0	0	0.0	
	4 or more weeks	6	18.8	6	75.0	1	25.0	
Pain intensity	Absence of pain	6	18.8	2	25.0	0	0.0	0.270
	Mild (1-3 pain score)	11	34.4	0	0.0	3	75.0	
	Moderate (4-5 pain score)	4	12.5	1	12.0	0	0.0	
	Strong (6-7 pain score)	7	21.9	4	50.0	0	0.0	
	Very strong (8-9 pain score)	4	12.5	1	12.5	1	25.0	
Regression of lesion	Complete	31	96.9	8	100.0	4	100.0	0.825
	Partial	1	3.1	0	0.0	0	0.0	
